# Mesenchymal Stem Cell-Derived Extracellular Vesicles as Non-Coding RNA Therapeutic Vehicles in Autoimmune Diseases

**DOI:** 10.3390/pharmaceutics14040733

**Published:** 2022-03-29

**Authors:** Olga Martinez-Arroyo, Ana Ortega, Maria J. Forner, Raquel Cortes

**Affiliations:** 1 Cardiometabolic and Renal Risk Research Group, INCLIVA Biomedical Research Institute, 46010 Valencia, Spain; omartinez@incliva.es (O.M.-A.); maria.jose.forner@uv.es (M.J.F.); 2 Internal Medicine Unit, Hospital Clinico Universitario, 46010 Valencia, Spain

**Keywords:** mesenchymal stem cells, extracellular vesicles, exosomes, non-coding RNA, microRNA, immunomodulation, autoimmune diseases

## Abstract

Autoimmune diseases (ADs) are characterized by the activation of the immune system against self-antigens. More common in women than in men and with an early onset, their incidence is increasing worldwide, and this, combined with their chronic nature, is contributing to an enlarged medical and economic burden. Conventional immunosuppressive agents are designed to alleviate symptoms but do not constitute an effective therapy, highlighting a need to develop new alternatives. In this regard, mesenchymal stem cells (MSCs) have demonstrated powerful immunosuppressive and regenerative effects. MSC-derived extracellular vesicles (MSC-EVs) have shown some advantages, such as less immunogenicity, and are proposed as novel therapies for ADs. In this review, we summarize current perspectives on therapeutic options for ADs based on MSCs and MSC-EVs, focusing particularly on their mechanism of action exerted through their non-coding RNA (ncRNA) cargo. A complete state-of-the-art review was performed, centralized on some of the most severe ADs (rheumatoid arthritis, autoimmune type 1 diabetes mellitus, and systemic lupus erythematosus), giving evidence that a promising field is evolving to overcome the current knowledge and provide new therapeutic possibilities centered on MSC-EVs and their role as ncRNA delivery vehicles for AD gene therapy.

## 1. Introduction

The incidence of autoimmune diseases (AD) is increasing worldwide, and some of the most severe, autoimmune type 1 diabetes mellitus (T1DM), systemic lupus erythematosus (SLE), and rheumatoid arthritis (RA), are estimated to affect at least 2–5% of the population in different regions of the world [1,2]. Many of these diseases develop around the 20–40-year age range, being more common in women than in men, with an enormous medical and economic burden due to their chronic course. ADs are characterized by activation of the immune system against self-antigens and can be classified as organ-specific (T1DM) or multiple systemic-involved conditions (SLE and RA), based on the affected organs [3]. In addition, disease courses are characterized by periods of aggressive autoimmune assaults followed by intervals of decreased inflammation and partial recovery of the affected tissues. In particular, autoreactive B- and T-cells trigger and drive multiple tissue destruction in the context of inappropriate local inflammation with the contribution of innate immunity [4]. The immune targets of T1DM, SLE, and RA are distinct; however, endoplasmic reticulum stress, reactive oxygen species, and pro-inflammatory and anti-inflammatory cytokines are shared mediators of tissue damage in these pathologies [5,6,7].

An enhanced understanding of the contribution of pro-inflammatory cytokines in the pathogenesis of certain ADs has led to the introduction of biologicals for their treatment [7,8,9]. However, biological agent treatment of ADs has several limitations, including high costs, immunogenicity with the development of neutralizing antibodies, along with side effects caused by their toxicity [10]. So far, there is no real and effective therapy for ADs, and conventional immunosuppressive agents such as methotrexate, steroids, and infliximab act only to alleviate symptoms [11]. Therefore, clinical scientists consider that alternative, more precise, and specifically targeted therapeutic strategies are urgently needed. Interestingly, several experimental and clinical investigations point to cell-based therapies by mesenchymal stem cells (MSCs) as a novel therapeutic option for ADs [12,13,14].

The immune regulatory and regenerative properties of MSCs provide new insights into AD treatment. It has been demonstrated that MSCs have the ability to regulate both innate and adaptive immune responses and can be used to treat RA, T1DM, and SLE [15,16,17]. MSCs can suppress T- and B-cell development and proliferation [18,19], promote the polarization of macrophages [20], induce more regulatory T-cells (Tregs) [21], reduce the maturation and cytotoxicity of natural killer (NK) cells [22], as well as impair dendritic cells (DCs) function [23]. In addition, growing evidence indicates that the predominant mechanism by which MSCs exert their effects is producing a large variety of paracrine, rather than cell-to-cell interaction, “secretome” mediators [24]. These soluble factors include cytokines, chemokines, messenger RNA (mRNA), growth factors, and so forth, the most important of which are extracellular vesicles (EVs), which can mimic MSC-based immunomodulatory and regenerative effects by delivering bioactive components, such as lipids, proteins, nucleic acids, and so on [25,26].

EVs are lipid-bilayer-enclosed nanoscale vesicles released by almost all cell types in both healthy and pathological conditions, which can be purified from various biofluids [27]. They play an essential role in cell-to-cell communication by transporting and delivering a vast variety of bioactive molecules, including proteins, lipids, mRNA, and non-coding RNAs (ncRNAs), such as long non-coding RNAs (lncRNA) and microRNAs (miRNAs), from parent cells to recipient cells [28]. MSC-derived EVs not only contribute to the restoration of damaged tissue and alleviate the systemic inflammatory response but also avoid the disadvantages of their original cells [29,30]. It is worth noting several concerns that have been raised regarding stem cell therapy including immune rejection, senescence, low cell survival, and the fact that genetic manipulation of MSCs can enhance the oncogenic potential of the cells [31]. In light of these observations, interest in the clinical applications of MSC-derived EVs (MSC-EVs) as drug delivery carriers, among other functions, has been increasing in recent years [30]. This review summarizes recent advances in the functional role of MSCs and MSC-EVs in three severe ADs (T1DM, SLE, and RA), with a special focus on their potential therapeutic effects as gene delivery vehicles of ncRNAs, providing a rationale for further research into MSCs and MSC-EVs in this field.

## 2. Properties of MSCs in Autoimmune Diseases

**Figure 1:**
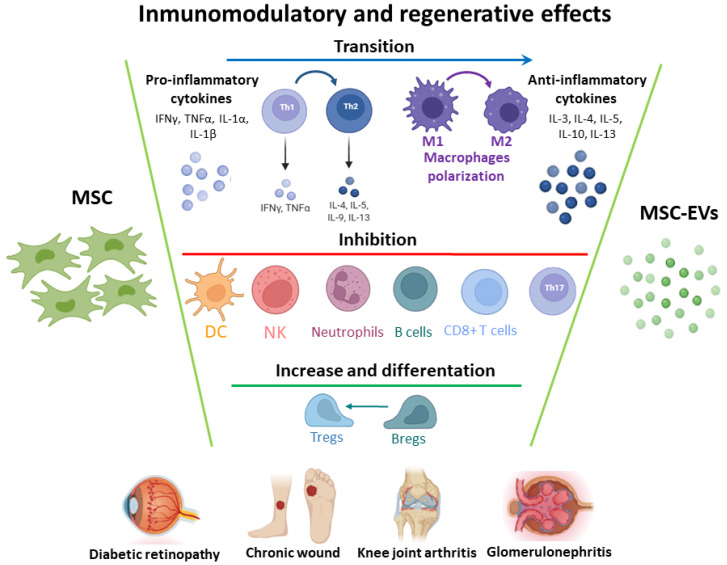
Immunomodulatory and pro-regenerative effects of mesenchymal stem cells (MSC) or MSC-derived extracellular vesicles (MSC-EVs) in autoimmune diseases. MSCs exert effects on T and B lymphocytes, natural killers (NK), dendritic cells (DC), neutrophils and macrophages by direct cell-cell interaction of EVs secretion, protecting and regenerating the damaged cells and mitigating the immune response. Breg: B regulatory cells; IFN: interferon; IL: interleukin; Th1: T helper 1 effector cells; Th2: T helper 2 effector cells; TNF: tumor necrosis factor; Treg: T regulatory cells. *Created mainly in biorender.com*.

MSCs are a primitive and heterogeneous population of non-hematopoietic multipotent cells that were discovered first as a fibroblast-like cellular population in the bone marrow (BM) [32,33]. MSCs were primarily isolated from BM, but they can be obtained from other human sources [34]. In 2006, the International Society of Cellular Therapy (ISCT) defined MSCs as cells with plastic adhesion and growth properties, with the expression of specific cell surface molecules, and with multipotent differentiation capacities under in vitro conditions [35]. MSCs are able to exert a range of biological functions, the most well-known being immunosuppressive and regenerative effects. Collectively, this suggests MSCs-therapy for treatment of ADs (Figure 1) [16,36].

### 2.1. Immunoregulatory and Immunosuppressive Potential

MSCs exert their immunomodulatory effects, high immunosuppressive ability, and low immunogenicity on both innate and adaptive immune cells through a wide diversity of mechanisms [16,37]. Thanks to these characteristics, they are extensively studied for their therapeutic advantages in different inflammatory and ADs. 

Multiple subsets of immune cells are present in the autoimmune milieu, and crosstalk between MSCs and key immune cell players impacts on inflammatory mediators in ADs. MSCs can regulate the inhibition of proliferation and differentiation of B-cells and T helper (Th)1, Th17 cells, induction of Treg activation, suppression of DCs maturation, promotion of the polarization of macrophages to M2, and inhibition of NK cell function [38]. MSCs also exert immunomodulatory roles through soluble factors such as prostaglandin–E2 (PGE2), IDO, NO, transforming growth factor–β (TGF-β1), and human leukocyte antigen-G5 [39,40].

The most significant effect of MSCs on T-cells is to inhibit the proliferation of Th17, Th1, and granulocyte-macrophage colony-stimulating factor-expressing CD4^+^ T cells. MSCs also induce Th2, an anti-inflammatory subtype [41]. A study by Ma et al. demonstrated that human umbilical cord MSCs (UC-MSCs) can reduce Th17 cell number via down-regulating RORγt, and up-regulating Foxp3 to augment the Treg percentage in RA [20]. Rashedi et al. reported that MSCs can indirectly induce CD4+ lymphocytes to differentiate into Treg cells or directly interact with Tregs through the Notch signaling [42]. In addition, BM-MSCs can also inhibit the production of inflammatory cytokines (TNF-α, IL-17, IL-6, IL-2, IFN-γ, and IL-9) via T-cells in RA [43], and MSCs restores the immune response by enhancing Treg/Th17 cell ratios and regulating TGF-β and PGE2 [44,45]. 

Besides, MSCs also inhibit B lymphocyte proliferation, differentiation, antibody production, and chemotaxis under inflammatory conditions [46], and favor regulatory B (Breg,) cell expansion. A recent study has implied that MSCs prevent proliferation, differentiation, and antibody secretion of B-cells through the CCL2-MST1-mTOR-STAT1-mediated metabolic signaling pathway [47]. Moreover, MSCs enhance increased Breg cells through the SDF-1-CXCR7 axis, maintaining immune tolerance and inhibiting inflammatory and immune responses [48]. Most interestingly, the suppressive activities of MSCs on B-cells also depend on the interaction between MSCs and T-cells [19,49]. 

Furthermore, MSCs affect the DC’s maturation by down-regulating co-stimulatory molecule expression and MHC class II [50]. Lu et al. suggested that MSCs attenuate lipopolysaccharide-induced acute lung injury by inducing the production of regulatory DCs (DCregs) via Notch signaling activation [51]. A recent study showed that the mechanism by which MSCs inhibit DCs may be achieved through the CD200/CD200R pathway, which acts mainly on the DC maturation process [52]. In addition, MSCs increase the ability of monocyte-derived DCs to polarize IL-17-/IL-10-producing T-cells via CTLA-4 [53]. Interestingly, human MSCs also express several ligands for activating NK cell receptors, and through the high-level expression of TGF-γ, IDO, and PGE2, decrease NK cell proliferation [54,55]. Interactions between MSCs and NK cells are necessary to reduce NK cytotoxicity. In addition, a recent study revealed differential crosstalk between MSCs and two highly cytotoxic NK lines, KHYG-1 and NK-92, and this fact should be taken into account when designing cell therapy protocols [23]. Finally, MSCs can reprogram the functions of the macrophage by driving macrophage differentiation into anti-inflammatory phenotype M2, inhibiting nuclear factor-κb/p65, altering their metabolic status via a PGE2-dependent mechanism, or activating signal transducer and activator of transcription 3 signaling pathways [56,57,58]. 

Taken together, these findings show MSCs to be involved in autoimmune disorders by influencing immune cell proliferation, differentiation, and function. Given the high plasticity of their immunomodulatory effects, research may be needed to determine the application of MSCs in treating ADs. However, the microenvironment influences the induction, increase, and maintenance of MSCs’ immunoregulatory role [59]. Blocking immune cell reprogramming and maintaining MSC roles in the immune microenvironment would provide new insights into the identification of valuable strategies for the biological treatment of ADs. 

### 2.2. Regenerative Properties

The value of MSCs in the application of regenerative medicine has been a focus of in-depth study in recent years [60,61], centered mainly on the reconstruction of fragile tissues, including the musculoskeletal system, nervous system, cornea, trachea, myocardium, liver, and skin [60]. 

The safety and efficacy of MSCs in the treatment of joint-related diseases and cartilage injuries have been continuously examined over the last few decades [62]. The promising qualities of MSC-based therapies could potentially provide effective and less-invasive procedures to repair articular cartilage defects [63]. First, MSCs can differentiate into chondrocytes [64]. Second, MSCs secrete a large number of trophic factors to promote angiogenesis, anti-fibrosis, anti-apoptosis, and other processes [64]. Moreover, novel bioactive 3D scaffolds, such as hydrogels [65] and electrospun scaffolds [66], provide an optimal 3D microenvironment for cartilage regeneration. Moreover, the biocompatibility of MSCs and hydrogels can facilitate MSC proliferation, differentiation, migration, and adhesion, potentiating the role of MSCs as a regenerative drug [65,67]. Injectable hydrogels allow minimally invasive treatment in cartilage repair [68]; thus, hydrogels loaded with MSCs and bioactive components are highly effective for repairing cartilage damage. Despite promising clinical trials [69,70], there are no commercially available products for MSC-based cartilage repair. 

MSC therapy may be a promising option for islet transplantation in T1DM, reducing immune reactivity and promoting vascularization, cell survival, and regeneration [71]. A study by Lee et al. demonstrated that multipotent stromal cells from BM promote the repair of pancreatic islets and renal glomeruli in diabetic mice [72]. Another study provided a fundamental understanding of MSC interactions in rescuing fulminant hepatic failure in pigs [73]. Moreover, several clinical trials showed that allogeneic MSC therapy is a potential option for the treatment of liver cirrhosis caused by ADs [74], as a means of enhancing liver transplantation results to induce tolerance after transplantation [75] or for improving histologic fibrosis and liver function in patients with alcoholic cirrhosis [76]. 

Recent progress has also been reported in skin regeneration, in which MSCs significantly improve wound condition and angiogenesis [77,78]. Besides, artificial scaffolds combined with MSC-based therapy offer a promising strategy to facilitate wound healing or the complete reconstruction of skin. A large number of different types of scaffolds, such as fibrin hydrogels [79], albumin [80], sericin hydrogels [81], boron oxygen-sensing nanoparticles impregnated into a polycaprolactone/chitosan layer [82], and curcumin nanoparticle-loaded collagen-alginate scaffolds [83], have been developed to support the role of MSCs as a regenerative strategy of defective skin. 

Finally, MSC therapy has been investigated for nerve regeneration, corneal repair, attenuating myocardial damage, and other musculoskeletal tissue outside bone and cartilage, with excellent outcomes [84,85,86,87].

Current evidence supports the use of MSCs for tissue regeneration. However, the use of MSCs to treat human diseases has raised several concerns, such as limited cell survival, senescence-induced genetic instability, loss of function, and immune-mediated rejection. Although preclinical studies demonstrate the possibility of MSC-based therapy in animal models, there are few reports of its clinical application. Further works including stem cell biology, biomaterials, and tissue engineering are crucial for MSC therapy. 

### 2.3. MSC-EVs as Cell-Free Therapy 

MSCs are not the only cells to show great immunomodulatory and regenerative potential; their secretome, including MSC-EVs as vehicles of paracrine factors, have demonstrated similar effects, which confer some advantages over their parent MSCs, representing a promising cell-free therapeutic alternative. 

As defined by the International Society of Extracellular Vesicles (ISEV), EVs are small, round-shaped phospholipid bilayer structures released into the intercellular space by most types of eukaryotic cells, and in many body biofluids [88]. EVs comprise a heterogeneous population including exosomes, microvesicles (MVs), and apoptotic bodies [89]. Exosomes are the smallest, ranging from 40 to 150 nm, originating from the endosomal pathway creating multivesicular bodies, and released by fusion with the plasma membrane. MVs, microparticles, or ectosomes are 100 to 1000 nm in size and are directly formed by blebbing and budding mechanisms from the plasma membrane; and lastly, apoptotic bodies (1000–5000 nm) are released from blebbing cells during the late stages of apoptosis [28,90]. The main role of EVs, which underpins their growing status as important players in physiological and pathological states, is to participate in intercellular communication to exchange biomolecules such as DNA, mRNA, lipids, proteins, and ncRNA [88,91]. Accumulating evidence has shown that specific miRNA profiles are diagnostic biomarkers and therapeutic targets in several ADs [92,93].

MSC-EV offers several different advantages over parent stem cells with regard to both regulatory approval and therapeutic development [94]. EVs are not subject to Food and Drug Administration (FDA) rules regulating the administration of living cells and do not possess carcinogenic potential themselves. In addition, EVs are highly stable and easily stored for long-term usage, and there is no evidence that EV doses can have divergent immunomodulatory influences [88,95]. Furthermore, EVs are also able to cross the blood–brain barrier, an advantageous characteristic for treating patients with central nervous system involvement, as occurs in ADs [96]. Finally, the application of EVs produced by genetically manipulated MSCs cells is not subject to additional regulation. According to ISEV, EVs will be considered biological medicinal products, so EV-based therapies would be considered in accordance with guidelines regulating medicinal products [97]. 

#### 2.3.1. Immunomodulatory Properties

Increasing research suggests that MSC-EVs possess similar immunomodulatory properties as MSCs [98]; MSC-EVs can also exert immunosuppressive effects on T-cells [99], B-cells [100], macrophages [101], DCs [102], and NK cells [103]. 

Similarly, MSC-EVs can inhibit T-cell activation and development by decreasing interferon-γ generated by CD4^+^ T cells [104,105], induce cell cycle arrest through the p27kip1/Cdk2 signaling pathway [106], and promote the differentiation of Th1 and Treg from naïve CD4^+^ T-cells [107]. MSC-derived MVs can induce apoptosis in activated T-cells and also boost the production of CD4^+^CD25^+^Foxp3^+^ Tregs [108], also by increasing IL-10 and TGF-β [109,110]. In B-cells, MSC-EVs reduced immunoglobulin production and inhibited B-cell proliferation and differentiation [111]. The immunosuppressive role of MSC-EVs in macrophages is also well-known. Hyvarinen et al. have shown that MSC-EVs down-regulate IL-23 and IL-22 and promote inflammation, enhancing the anti-inflammatory phenotype of regulatory macrophages [112]. EVs derived from MSCs can suppress pro-inflammatory phenotype (M1) macrophage activation but promote anti-inflammatory phenotype (M2) macrophage activation [101,113]. Accumulating data indicate that MSC-EVs inhibit NK cell proliferation by regulating TGF-β [114]. In addition, most MSC-EV immunoregulatory roles are regulated by carrying various “messengers”: DNA, mRNA, proteins, lipids, and ncRNAs [89,115]. In summary, these findings provide evidence for an immunoregulatory effect of MSC-EVs. 

#### 2.3.2. Regenerative Effect

The regenerative action of MSC-EVs has been well documented in previously published studies [116,117]. A Zhang et al. study demonstrated, for the first time, the efficacy of human embryonic MSC exosomes in cartilage repair [118]. Recent research showed that MSC-EVs not only promoted proliferation and migration of human osteoarthritis (OA) chondrocytes but also maintained the chondrocyte matrix [119]. Moreover, MSC-EVs were internalized by OA chondrocytes, repealed TNF-α adverse effects, and promoted cartilage regeneration [120]. For other musculoskeletal tissue outside cartilage, Chen et al. tested the ability of MSC-EVs to repair tendon damage, obtaining a greater tenocyte proliferation and migration, together with an improvement of the mechanical stress tendon [121]. Nakamura et al. showed that MSC-derived exosomes (MSC-exos) promote muscle regeneration by enhancing myogenesis and angiogenesis [122]. 

Currently, numerous studies show that MSC-EVs may positively modulate different phases of the wound-healing process [123]. A recent study by Ren et al. showed that MSC-EVs therapy of skin wounds in mice produced an increase in proliferation and vascularization of endothelial cells and increased collagen deposition, resulting in accelerated healing in untreated wounds [124]. In addition, hypoxic MSC-exos applied to skin wounds in a diabetic rat model were more effective in inducing angiogenesis and promoting wound healing than the unconditioned MSC-EVs [125]. Another potential advantage of MSC-EVs is their scarless wound healing capability. In a recent study, Wang et al. accelerated wound closure in a full-thickness skin wound mouse model, inducing the expression of genes encoding ECM proteins and increasing the proliferation and migration of dermal fibroblasts [126]. This EV collagen induction result is promoted in the early stages of wound healing, while it inhibits collagen biosynthesis at later stages [127]. Besides this, MSC-EVs promoted the proliferation and migration of skin keratinocytes, with the upregulation of proliferative marker genes such as cyclins, as well as fibronectin [124]. Finally, the exogenous cell-free approach of using MSC-EVs therapeutically has shown promising results in attenuating neurodegenerative disorders [128], myocardial damage [129], and corneal damage [130], among others. 

**Figure 2:**
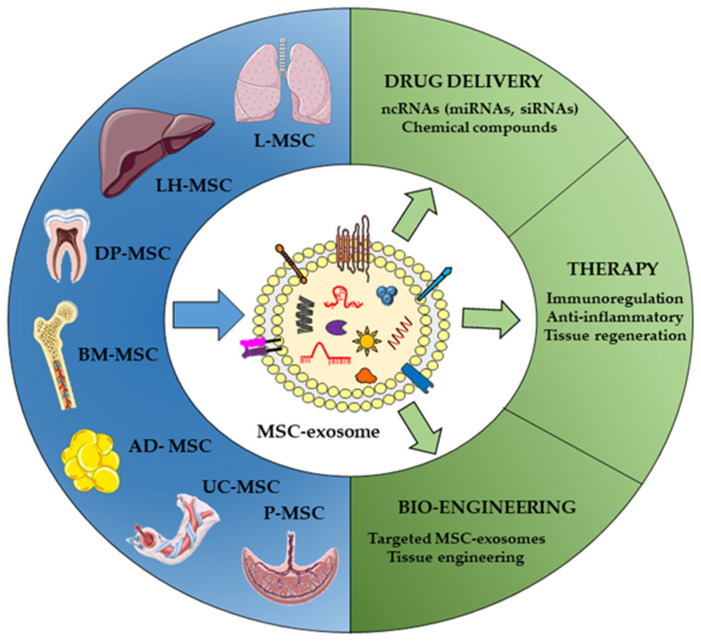
Origin and therapeutic purposes of mesenchymal stem cells (MSC) and MSC-derived exosomes (MSC-Exos). MSCs are found in many tissues, such as lung (L), liver (LH), dental pulp (DP), bone marrow (BM), adipose tissue (AD), umbilical cord (UC) and placenta (P). MSC-Exos play therapeutic roles as drug delivery systems (targeted MSC-exos) for immunomodulation, an-ti-inflammatory effect and tissue regeneration and engineering. *Created mainly in biorender.com*.

Recent studies have demonstrated that MSC-EV-ncRNAs, and miRNAs overall, exert significant immunoregulatory [131,132] and regenerative effects [133]. In the following sections of this review, we outline the therapeutic role of ncRNAs packaged into MSC-EVs in ADs, focusing on examples such as drug delivery systems (Figure 2).

## 3. Key Points in MSC EVs as Non-Coding RNA Delivery Vehicles

### 3.1. Different Sources of MSCs

Although the BM was the first described source of MSCs [134,135], they have subsequently been isolated from other adult tissues such as placenta, umbilical cord, dental pulp, and adipose tissue. The most frequent sources of MSC for therapeutic use have been BM-MSC, adipose tissue (AD-MSC), and fetal tissues (amniotic membrane and placenta (P-MSC), and UC-MSC tissue or blood) [136,137]. MSCs differ in terms of their proliferation activity, multilineage capacities, overall gene expression profile, and biological functions [138,139]. BM-MSCs were the best-described source of MSCs used in clinical trials, although their biological capacities decrease with age and vary according to donor sources [139,140]. Painful invasive procedures are required to obtain BM samples and there is a risk of viral exposure. AD-MSCs can be easily obtained via subcutaneous lipoaspiration and isolated from stromal vascular fractions and can yield up to 500-fold more MSCs than BM [141]. In a recent study, Menard et al. showed that AD-MSC exhibited a strong inhibition of immune response and low immunogenicity, supporting the use of adipose tissue as an important source for clinical applications in immune-mediated diseases [142]. Human MSC from neonatal tissues such as umbilical cord, placental decidua, and amniotic and chorionic membranes, are good options for cell therapy due to their biological characteristics as immature cells [143]. Of interest, UC-MSC may be less immunogenic and display a unique gene expression profile, modulating processes such as immunomodulation, angiogenesis, wound healing, apoptosis, antitumor activity, and chemotaxis, compared to BM-MSC [144,145]. Another study compared the immunophenotype, proliferative potential, multilineage differentiation, and immunomodulatory capacity of MSCs derived from BM, adipose tissue, placenta, and umbilical cord blood, concluding that BM-MSCs and AD-MSCs represent the optimal stem cell source for tissue engineering and regenerative medicine [146]. In 2014, Rolandsson et al. demonstrated that MSC isolated from central and peripheral transbronchial biopsies of lung-transplanted patients were found perivascularly and enriched in CD90 and CD105 surface proteins and lung resident MSC (LR-MSC) [147]. Later, LR-MSC were phenotypically characterized and compared with BM-MSCs, highlighting their unique phenotype and gene expression pattern [148]. The liver is a novel reservoir of stem cells. Compared with MSC from other tissues, liver-derived human MSCs (LHMSCs) possess similar morphological properties, immune functions, and differentiation capacities [149]. However, LHMCSs are different in their surface proteins and biological functions. In 2009, a group of investigators ranging from basic biologists to clinical hepatologists first defined standardized methods to assess stem/progenitor cells or their hepatic lineage-committed progeny that could be used for cell therapy in liver disease [150]. Finally, a recent preliminary study described the basic characteristics and biological functions of menstrual MSCs and their derived EVs. They also demonstrated the therapeutic potential of small EVs in fulminant hepatic failure, myocardial infarction, pulmonary fibrosis, prostate cancer, cutaneous wound, T1DM, aged fertility, and potential diseases [151].

### 3.2. Non-Coding RNA Cargos

The effect of MSC-EVs on receptor cells depends on their origin but also mainly on their content. Apart from known cargos such as lipids, proteins, and DNA, ncRNAs have emerged as a great source of potential EV-linked therapies due to their mechanism of action and the variety of exerted effects, including the regulation of biological processes, modification of cellular phenotype, and mediation in intercellular communication [152,153]. ncRNAs have been shown to act at all levels of gene regulation, including transcription, mRNA stability, and translation [154]. In the progression of therapies based on EV transfer, studies are increasingly focused on ncRNAs cargos, either using those present biologically in EVs or developing techniques to chemically load them inside EVs for targeted delivery. In addition, RNA sequencing studies have enabled the study of MSC-EV content and have convincingly been shown to be enriched in non-coding cargos [155,156].

Widely studied as EV cargos, miRNAs carry out multiple functions in target cells. Biologically, they interact with mRNAs regulating transcription and also act at the posttranscriptional level. Their expression has been demonstrated to change between physiological and pathological states, which makes them ideal targets for disease diagnosis and therapy. Indeed, most authors report their usefulness for therapeutic approaches using MSCs [157,158]. In a recent study by Xiong et al., exosomal miR-21 from P-MSCs attenuates senescence by regulating the PTEN/PI3K-Nrf2 axis and elevating the antioxidant capacity [159]. Autoimmune and inflammatory diseases and cancer are the most common focuses of study in this field; for instance, in an OA in vitro model, it was shown that miR-129-5p in human synovial MSC (HS-MSC)-derived exosomes decreased the inflammatory response and apoptosis of chondrocytes by targeting the 3′UTR of HMGB1 [160].

Small interfering RNAs (siRNAs) are small RNA molecules whose regulation mechanism consists of silencing gene expression by either transcription suppression or by triggering sequence-specific degradation of target mRNAs [161]. The therapeutic use of siRNAs enclosed in MSC-EVs is mainly based on their artificial loading for specific delivery to target cells. This strategy offers certain advantages over the use of viral or physical and chemical vectors for gene therapy, as, despite representing great advances in the field, they still have certain limitations such as immunogenicity, systemic inflammatory response, toxicity, and failure to deliver high doses in vivo [162,163,164]. These limitations are resolved with the use of EV-siRNAs, since engineered exosomes are able to effectively deliver siRNAs such as siKRAS or siPLK1 to target cells and knockdown their expression, inhibiting lung and bladder tumor growth, respectively [165,166]. Particularly, the use of hybrid nanovesicles composed of exosomes and liposomes has shown good results, combining the benefits of both systems to achieve effective cargo delivery [167]. MSC-EV-based therapies with siRNAs are poorly developed, yet some studies have demonstrated their effectiveness [168,169]. Both in vitro and in a spinal cord injury disease model, Huang et al. showed that MSC-exos loaded with CTGF siRNA produce decreased inflammation and neuronal apoptosis, also reducing glial scar formation and positively activating factors for locomotor recovery [170]. More surprisingly, another study in spinal cord injury demonstrated that the intranasal delivery of phosphatase and tensin siRNAs from MSC-exos promoted a complete recovery from injury, enhancing axonal growth and neovascularization [171]. Despite the paucity of research available as yet, promising results are being obtained for siRNA therapies in this field, with EVs as optimal delivery carriers.

LncRNAs are a diverse class of ncRNAs longer than 200 nt that engage in key biological processes through epigenetic, transcriptional, and post-transcriptional regulation [172,173]. Although their structural and functional characterization is not yet well defined, lncRNAs account for a large percentage of the transcriptome, and novel types are continuously discovered and functionally described. LncRNAs have been proposed not only as biomarkers but also as potential therapeutic elements, given their broad spectrum of described functions such as epigenetics, nuclear import, alternative splicing, and their ability to interact with miRNAs through several mechanisms [172,174,175]. In the context of ADs and inflammatory diseases, lncRNAs are expressed in and regulate various immune system cell types, and their altered levels have been related to autoimmunity and disease features [176,177,178]. Consequently, a strategy based on lncRNA-EV-mediated delivery is emerging as a new frontier in therapy development [179]. Proof of this is the use of MSC-exos with the lncRNA Klf3-AS1 for OA treatment, which promoted cartilage repair and chondrocyte proliferation in an OA rat model [180]. Similarly, lncRNA SNHG7 transported inside exosomes derived from MSCs curbed the endothelial–mesenchymal transition and tube formation in diabetic retinopathy through interaction with miR34a-5p, demonstrating the valuable role of lncRNAs for miRNA regulation [181].

Not as extensively studied as other ncRNAs, circular RNAs (circRNAs) are abundant and stable covalently closed single-stranded RNAs without 5′ and 3′ ends [182,183]. The lack of these ends makes circRNAs resistant to exonuclease degradation, and thus more stable than most linear ncRNAs. CircRNAs have been shown to regulate the transcription of miRNA target genes acting as miRNA sponges, interact with RNA binding proteins, and also participate in mRNA stability and protein translation [184,185]. The involvement of circRNAs has been reported in several diseases, mostly in cancer, thus they are increasingly proposed as EV therapeutic cargos. In a recent study on pancreatic cancer, exosomal circ_0030167 from BM-MSCs prevented cell invasion, migration, and proliferation, acting as a sponge for miR-338-5p [186]. Moreover, therapeutic use with MSC-EVs also extends to autoimmune and inflammatory diseases [187,188].

Finally, other non-coding cargos of EVs include Piwi-interacting RNAs (piRNAs), small RNAs that interact with PIWI proteins to silence genetic elements such as transposons, with functions ranging from gene expression regulation to viral defense [189]. PiRNAs have revealed key implications in cancer and neurodegenerative disorders, and have been proposed as markers in diagnostic, prognostic, and treatment aims [190,191,192]. Although their presence in MSC-EVs has been demonstrated [156], no studies using piRNAs as therapeutic cargos in MSC-EVs have been conducted to date.

### 3.3. Separation Methods

EVs isolation and purification constitute the first step to use them as therapeutic agents. Despite interest in the field during recent years, there is still a need for well-designed protocols with consensus among the scientific community for the isolation and characterization of EVs. The optimal isolation method will depend mainly on the quantity and type of initial sample, the amount of EVs present in them, and their final use.

Differential ultracentrifugation (UC) and density gradient UC are both considered gold standard methods for EV isolation and have been in use for decades. Traditional UC protocols employ centrifugal force, which mediates sequential centrifugations with increasing velocity to precipitate different vesicles according to their sedimentation coefficient rate. UC allows large sample volume processing (urine and cell medium) but requires specific and expensive equipment [193]. To improve the purity and fractionation of EV subpopulations, density gradient UC separates vesicles using a pre-constructed decreasing gradient medium, usually made of sucrose, iohexol, or iodixanol. Combining both methods could be an interesting alternative when aiming to process large volumes with high purity [194].

Size-based methods, ultrafiltration, and size exclusion chromatography (SEC) have been extensively used to separate mixtures with molecules of different sizes. For ultrafiltration, a semipermeable membrane is used, usually made of cellulose, polyethersulfone, or Hydrosart, with a defined pore size between 10 and 100 KDa. This technique is also employed for the concentration of samples isolated by other methods such as UC, and can greatly enrich exosome concentration at a low cost but with low purity [195,196]. Conversely, SEC uses columns with a stationary phase, commonly a porous resin such as sepharose. Dissolved in a mobile phase, samples will migrate and separate along the resin based on their differential elution profile. The smallest particles, such as exosomes, penetrate the porous resin and are eluted after larger particles, also with minimal structural damage [197,198].

Polymer-based precipitation is based on the use of highly hydrophilic polymers (Polyethylene glycol (PEG)), which interact with water molecules surrounding the exosomes to create a hydrophobic microenvironment, resulting in exosome precipitation with low-speed centrifugation [199,200]. However, certain aggregates and proteins can sometimes be precipitated along with exosomes, decreasing the purity of the sample. To solve this problem, exosomes can be immunoprecipitated by incubating the sample to specific exosome surface markers with antibody-coupled magnetic beads [201,202]. Indeed, affinity precipitation methods open up the possibility of isolating EVs from specific cells/tissue, which could be an interesting option. Nonetheless, this methodology is still the most expensive due to the use of antibodies, and the EV marker panel needs to be improved.

Finally, microfluidic technology specifically captures and isolates exosomes, combining immunoaffinity, size, and density [203]. Through the use of chips with embedded antibodies, small sample amounts can be loaded to isolate exosomes. This methodology combines new advantages such as efficient and fast processing with high purity [204]. Microfluidics has promise, but the method is still in need of greater standardization.

The validation of isolated EVs is recommended before their use in order to ensure purity and experimental accuracy. The characterization of EVs allows us to determine their concentration, confirm the absence of contaminating proteins or organelles, and identify the cell of origin and cargo. Several techniques are employed for this purpose, such as nanoparticle tracking analyses (NTA) or dynamic light scattering (DLS) to assess the size and number of particles, transmission electron microscopy to confirm EVs’ morphology, and the well-known Western blot to evaluate the presence of contaminant proteins.

### 3.4. Exosome Administration Routes

Selecting the most suitable exosome administration route, including the dose administrated, and considering the stability, biodistribution, and toxicity of delivered exosomes is one of the keys to achieving successful cell-free therapy. The most commonly used administration routes employed in in vivo assays are described in the following subsections.

Despite being the most popular administration route for its ability to reach the whole organism, systemic injection presents some disadvantages, such as the accumulation of exosomes in non-targeted organs, commonly the liver, spleen, and lungs, and quick clearance from the organism [205]. In the context of ADs described in this review, numerous studies have employed this technique. In cutaneous affections such as T1DM-derived ulcers, subcutaneous injection of exosomes is an effective therapy. Several studies have described the potential application of exosomes to improve wound healing after cutaneous administration of injured areas [206,207,208]. Regarding skin administration methods, injection into the dermis, known as intradermal injection [187,208], and topical application [209,210] are other simple options to treat skin affections. Intraperitoneal delivery of exosomes is another administration route, with wide exosome distribution potential [211,212]. In 2018, Chen et al. proved that BM-MSC exosomes enriched in miR-150-5p suppress VEGF and MMP14 expression, resulting in negative regulation of IL-β, TNF-α, and TGF-β, which suppresses the proliferation and migration of fibroblast-like synoviocytes (FLS), angiogenesis, and alleviation of inflammation [213].

Intranasal administration is an attractive option when the objective is to cross the blood–brain barrier, avoiding exosome breakdown in other organs and favoring accumulation in the brain, resulting in better neuroprotective effects [171,214,215]. Oral treatment is an easier and non-invasive route, although the delivered exosomes must survive passage through the intestinal tract. In the context of ADs, 2015 was the first time that the oral administration of exosomes isolated from bovine milk had the potential to delay the onset of RA, decreasing cartilage injury, BM inflammation, and the reduction of MCP-1 and IL-6 levels [216].

Additionally, other options for exosome delivery are suitable for different conditions to be treated. Intra-articular injection next to the damaged tissue has been used for RA, resulting in more effective tissue regeneration due to the direct application of the therapeutic agent to the damaged area [217,218]. Likewise, subconjunctival, intravitreal, and intraocular administration, despite being invasive and disturbing methods, have been employed to treat retinal diabetes complications with positive results [219,220,221].

## 4. Therapeutic Effects of ncRNA in MSC EVs in Autoimmune Diseases

### 4.1. Rheumatoid Arthritis

RA is a chronic inflammatory AD that affects 0.5–1% of the general population, imposing a significant burden on the social and economic system. Among its principal features, the most frequent include hyperplasia of the synovial membrane, infiltration of inflammatory cells, bone and cartilage progressive destruction, and multiple organ involvement [222], leading to a worsening in life quality. Unfortunately, there is no cure for RA, making the discovery of new effective treatments to attenuate pain and stop further damage imperative.

The above-described anti-fibrotic and anti-inflammatory beneficial properties of MSCs have been also demonstrated in RA, such as their ability to improve RA progression by regulating memory T-cell responses [20,223,224]. Given their benefits, the use of MSCs-EVs as a cell-free therapeutic tool for the treatment of RA has become an interesting field of study during the last years. In this context, EVs have been associated with the propagation and attenuation of joint inflammation and destruction [225]. In a model of collagen-induced arthritis (CIA), Consenza et al. described, for the first time, that MSC-EVs, especially exosomes, promoted a reduction of disease signs by reducing plasma blast cells and increasing B-cells secreting IL-10 [226]. Furthermore, human UC-MSC-derived EVs can also inhibit the expression of IL-17 by down-regulating Th17 cells and increasing the proportion of Treg cells in a dose-dependent manner [20].

The therapeutic properties of EVs depend mostly on their cargo, among which miRNAs are the most predominant and well-known (Table 1). Consistently, MSC-exos enriched in miR-320a mimic significantly suppressed RA-FLS activation, migration, and invasion in vitro and diminished arthritis and bone damage in mice with CIA [227]. Additionally, miR-34a and miR-192-5p in EVs from BM-MSCs have been linked to the reduction of inflammation via the cyclin I/ATM/ATR/p53 axis [228] and to a delay in the inflammatory response [218]. Likewise, the overexpression of miR-124a in BM-MSC-derived EVs inhibited the proliferation and migration of the FLS cell line and promoted apoptosis of these cells during co-incubation [229].

**Figure 3:**
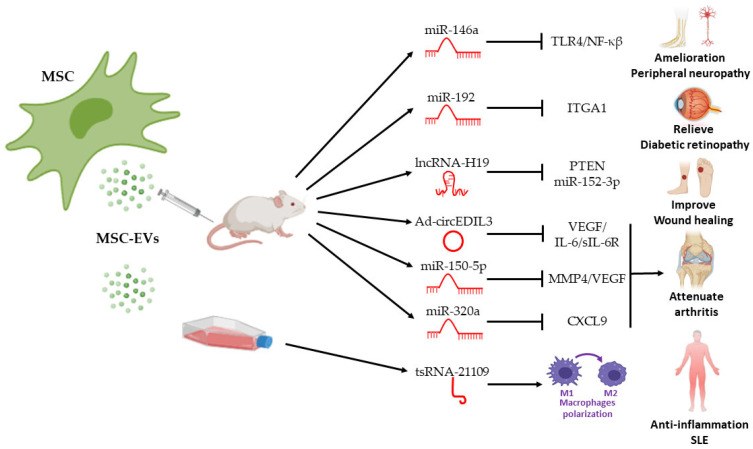
Summary of protective, regenerative or immunomodulatory capabilities of MSC-derived extracellular vesicles (MSC-EVs) administered in experimental models of different ADs. Several studies on non-coding RNA (miRNAs, lncRNA, circRNA and tsRNA) have demonstated their effects and mechanisms as therapeutic systems to ameliorate diabetic peripheral neuropathy, relieve diabetic retinopathy, improve wound healing of diabetic ulcers, attenuate arthritis and exert anti-inflammatory effect in systemic lupus erythematosus (SLE). Arrows indicate activation or induction, T-bars indicate inhibition. Circ: circular RNA; CXCL9: Chemokine (C-X-C motif) ligand 9; IL: interleukin; ITGA1: Integrin Subunit Alpha 1; lncRNA: long non-coding RNA; miRNA: microRNA; MMP4: matrix metalloproteinase 4; MSC: mesenchymal stem cells; PTEN: Phosphatase and tensin homolog; TRL4: Toll-like receptor 4; tsRNA: transfer RNA-derived fragments; VEGF: vascular endothelial growth factor. *Created mainly in biorender.com*.

Besides miRNAs, other ncRNAs such as lncRNAs [230,239,240] and circRNAs [187] have been reported to exert therapeutic effects in RA. As an example, MSC-exos lncRNA HAND2-AS1 re-expression suppressed proliferation, motility, and inflammation and triggered apoptosis in RA-FLS activation through the miR-143-3p/TNFAIP3/NF-kappaB pathway [230]. Recently, Zhang et al. demonstrated that the injection of both synovial mesenchymal cell (S-MSC)-derived exosomes or exosomes overexpressing circRNAs (Ad-circEDIL3) reduced synovial VEGF and consequently ameliorated arthritis severity in the CIA mouse model, by targeting the circEDIL3/miR-485-3p/PIAS3/STAT3/VEGF functional module in RA [217] (Figure 3).

Besides their inherent cargo, EVs can be loaded with several cargos or chemical drugs of interest and serve as biological delivery tools; these EVs can also be designed to target specific tissues, which opens up the field to develop more efficiently engineered EVs. Studies of this type are still scarce in RA disease, where, to date, only one study, developed by Topping et al., has been published. This research identified a specific anti-reactive oxygen species-collagen type II (Anti-ROS-CII) antibody able to target damaged arthritic cartilage and employed EVs isolated from human neutrophils with intrinsic immune regulatory functions and cartilage penetration as a vehicle to deliver Anti-ROS-CII into injured joints. The incorporation of Anti-ROS-CII into EVs by sonication and their consequent systemic administration revealed the ability to penetrate injured tissue while maintaining antibody activity, which accelerates the attenuation of clinical and synovial inflammation [241].

### 4.2. Type I Diabetes Mellitus

Diabetes mellitus is a highly prevalent metabolic disease with an elevated economic cost, mostly accompanied by cardiovascular complications and comorbidities. Two main types of diabetes have been defined: Type 1 (T1DM), which is autoimmune related, and type 2. T1DM is a chronic and complex disease recognized as an autoimmune disorder, characterized by long-term hyperglycemia due to permanent loss of insulin production as a consequence of impaired immune tolerance to β-cell antigens and pancreatic β-cell destruction [242,243]. The pathogenesis of T1DM is multifactorial and has been related to genetic susceptibility, which predisposes individuals to abnormal immune responses to pancreatic islets; however, 85% of patients have no family history of diabetes, so additional factors such as environmental ones must be involved in predisposition to the disease [244]. Inflammatory infiltrates are present in the islets of Langerhans, where diverse immune cells (CD8^+^ T cells, B cells, CD4^+^ T cells, NK cells, and macrophages) contribute differentially to defined pathogenic aspects of T1DM and to generating and maintaining the autoimmune response that characterizes the disease [245].

Exosomes exert two opposite effects, beneficial and detrimental, upon T1DM. Firstly, their ability to interact with immune cells such as T- and B-cells through their cargo make them immune system activators that can promote pancreatic β-cell apoptosis or induce inflammatory effects, conferring a pathogenic role in the onset of T1DM [246,247]. Conversely, recent advances attribute valuable properties for exosomes, showing them to be excellent candidates for T1DM treatment due to their modulatory effects on the immune cell response [212,231].

In this regard, developing MSC-derived exosomal therapies has important advantages in T1DM, since MSCs have demonstrated profound immunomodulatory effects and a potent regenerative capacity, as stated above [248,249]. In line with this, a randomized controlled trial developed several years ago in T1DM patients showed the potential of MSCs in preserving β-cell function, where patients were treated with autologous MSCs, obtaining promising results for their use in humans [250]. After years of accumulative research and studies, EVs derived from various types of MSCs have emerged as key players in T1DM therapy [212]. Recently, MSC-EVs showed a marked attenuation of the immune response by inhibiting antigen-presenting cell activation and suppression of Th1 and Th17 cell development [251]. Furthermore, exosomes from menstrual-blood-derived MSCs were intravenously injected in STZ diabetic rats, producing islet regeneration through pancreatic and duodenal homeobox 1 pathways [15]. One of the latest studies also revealed the attenuation of diabetes in a T1DM mice model by using exosomes from UC-MSCs. This study showed that exosome administration produced an increase in insulin production and an improvement in histological structure, showing β-cell regeneration [252]. Other studies investigated the effect of MSC-EV therapies in diabetes-induced cognitive impairment, in which exosomes derived from BM-MSCs revealed improved cognitive capacity in STZ mice, reversing neuronal and astroglial damage [253].

Exploring the use of MSC-EVs for therapy in more depth, a more precise and well-known mechanism of action of EV-mediated treatment is focused on their cargos [254]. During the last three years, increasing studies have described several exosomal ncRNA cargos from MSCs, mostly miRNAs, mediating the reduction of inflammation [219], promoting β-cell proliferation [255], or ameliorating different diabetic complications such as erectile dysfunction [232], peripheral neuropathy [231], cognitive impairment [256], diabetic retinopathy and retina degeneration [181,220,221,233], or diabetic nephropathy [234,235]). In a recent study by Chen et al., exosomal miR-21 from human UC-MSCs protected β-cells from hypoxia-induced apoptosis by inhibiting p38 MAPK signaling, alleviating ER stress, and improving cell survival [236]. Other ncRNAs, such as the MSC-derived exosomal lncRNA SNHG7, have demonstrated a relevant role in diabetic retinopathy. LncRNA SNHG7 reduces the endothelial–mesenchymal transition and tube formation in cell culture of retinal microvascular endothelial cells inhibiting the miR-34a-5p-XBP1 axis [181] (Figure 3).

Certain novel therapies involve engineering EV cargo in MSCs to enhance their beneficial effects or for specific targeting. An example of this is miR-146a for diabetic peripheral neuropathy treatment, transfected in BM-MSCs to enrich exosomes with this miRNA compared with natural exosomes, showing attenuation of diabetic-induced endothelial dysfunction, improvement of neurovascular function, and exerting immunosuppressive effects greater than those observed in naturally secreted exosomes [237].

One serious complication of diabetes for which effective treatment is lacking is the formation of chronic foot ulcers and delayed wound healing, which affects 15–25% of diabetic patients and increases the risk of gangrene, amputation, and death [257,258]. Vascular insufficiency is one of the principal causes of chronic non-healing cutaneous wounds, although compromised immunological status, advanced age, and chronic mechanical stress also contribute to poor wound healing [259]. Modulating the inflammatory response could represent a promising strategy to promote proper wound healing. Regenerative medicine strategies using MSCs have good potential as alternative approaches for chronic inflammation treatment. Several studies have shown the ability of MSCs to relieve inflammatory responses and accelerate skin wound healing [260,261]. Beyond this, MSC-EVs and their cargo have shown beneficial effects in preclinical animal studies for regeneration processes of diabetic foot ulcers, intervening in inflammation, angiogenesis, re-epithelialization, and remodeling stages [125,209]. An example of their regenerative power is the study from Yang et al. in which they design an engineered combination of human UC-MSC-exos and Pluronic F-127 hydrogel to improve wound healing in diabetic rats, revealing an accelerated wound closure rate, enhanced regeneration of granulation tissue, and up-regulated expression of VEGF and TGFβ-1 [210]. In addition, recent studies have shown that lncRNA H19 shuttled by MSC-exos or by mimetic nanovesicles has a potent effect on wound healing [208,262]. Specifically, lncRNA H19 prevents apoptosis and inflammation of fibroblasts by inhibiting miR-152-3p and promoting PTEN expression [208].

### 4.3. Systemic Lupus Erythematosus

SLE is a complex multisystemic AD extremely heterogeneous with a wide spectrum of different combinations of clinical manifestations, accumulation of laboratory and immunological abnormalities, and a variable disease progression and outcome. Using, for the most part, the American College of Rheumatology criteria (1982 or 1997), overall incidence rates for SLE vary from approximately 0.3–23.7 per 100,000 person-years, while prevalence rates have ranged from 6.5–178.0 per 100,000 [263]. The etiology and pathogenesis of SLE are accompanied by immune disorders including abnormal proliferation, differentiation, activation, and dysfunction of immune cells. Chronic inflammation causes tissue and organ damage, with lupus nephritis (LN) being the most common and serious manifestation in SLE [264,265]. Determining an SLE patient-tailored preventive strategy, therefore, remains an urgent problem to be resolved.

Current treatment strategies for SLE are focused mainly on controlling and mitigating activity flares, and MSC transplantation is regarded as a novel SLE treatment. Several studies have established that MSCs can attenuate the adverse effects of immunosuppressive drugs [266,267] and that allogeneic MSCs transplantation has brought new hope of curing severe SLE patients [268,269]. Chun et al. have demonstrated that transplantation of MSCs ameliorates SLE and upregulates B10 (IL-10-producing regulatory B) cells through TGF-β1 knockdown [270]. In addition, Liu et al. have confirmed that the transplantation of human-placenta-derived MSCs alleviates renal injury and reduces inflammation in a mouse model of LN [271]. Another recent study indicates that the transplantation of AD-MSCs could significantly inhibit autoimmune progression in MRL/lpr mice, and the efficacy of AD-MSCs was comparable to that of cyclophosphamide [272]. Yuan et al. showed that the transplantation of allogenic UC-MSCs induced FLT3L and CD1c + DCs in SLE patients, suppressing inflammation in lupus by up-regulating tolerogenic DCs [273]. Moreover, Choi et al. showed that MSC transplantation restored miRNA expression-associated lupus disease and Th1/Th2 ratios in a murine model of SLE [274]. Most importantly, mounting clinical studies have shown that allogenic AD-MSC transplantation was associated with good safety and efficiency in reducing disease activity and urine protein excretion [275]; however, the greatest proteinuria improvement was observed at 1 month and 6 months on disease activity indexes [268]. Moreover, more than a single dose of AD-MSCs may thus be required to maintain long-term remission of LN [276].

MSCs have immunomodulatory effects, mainly through cell-to-cell contact and through MSC-EV as a paracrine pathway [16]. EVs cargoes such as DNAs, mRNAs, miRNAs, lncRNAs, and circRNAs have been established to play crucial roles in regulating autoimmunity and are associated with SLE activity [92,277]. Accumulating evidence has implied that MSC-EV-derived ncRNAs play an important role in the pathogenesis of inflammatory and ADs [16,25,278]. Several recent studies have elucidated that exosome-deriving miRNAs, circRNAs, and other ncRNAs play important roles in SLE pathogenesis [92,176,279]. Although MSC therapies have shown promise in models of SLE-related pathologies, the function and mechanism of MSC-exos are still unclear. Eirin et al. identified the potency of EVs isolated from AD-MSCs for ameliorating renal injury. Intrarenal delivery of MSC-EVs decreased renal inflammation, increased the number of reparative macrophages, and up-regulated the expression of IL-10, suggesting that anti-inflammatory properties underpin the protective effects of EVs [280]. Another study demonstrated that human MSCs-exos attenuate alveolar hemorrhage through increasing M2 macrophage polarization in lupus mice [281]. In addition, a recent study by Dou and colleagues confirmed that MSC exosomal tsRNA-21109 alleviates SLE by inhibiting macrophage M1 polarization [238] (Figure 3). Moreover, LN is the most common and severe organ injury in SLE. Several works showed ncRNAs from MSC-exos as encouraging therapy for renal damage. Cao et al. demonstrated that MSC-exos ameliorates ischemic acute kidney injury and promotes tubular repair by targeting cell cycle arrest and apoptosis through the miR-125b-5p/p53 pathway [282].

## 5. Conclusions, Challenges, and Limitations Associated with MSC-EVs and MSC-EVs ncRNA Cargos

In recent years, the field of MSC-based therapies for AD has experienced a revolutionary change in perspective, thanks to the use of MSC-EVs, which overcomes the limitations observed in the use of MSCs but conserves their high capacity for immunomodulation and regenerative properties. The use of MSC-EVs offers several advantages: As a cell-free therapy, they provide stability and safety, avoiding tumorigenesis, genetic mutability, and immunogenicity compared to their parent MSCs, and allowing several modifications to their surface and cargo, which make them a promising potential treatment for ADs (Table 2). Furthermore, studies are increasingly focusing on EVs cargo for the development of targeted therapies, revealing the mechanisms by which MSC-EVs exert their beneficial effect. In this sense, ncRNAs are the gold players to become a powerful strategy since they regulate many biological processes and mediate intercellular communication, regulating gene expression.

Nevertheless, the use of MSC-EVs and their ncRNA cargos is still in the initial research and development stage and faces major obstacles and limitations. A principal challenge to overcome is the optimization of methods for MSC-EV characterization, high-scale production, and purification, with generalized and standardized protocols. Moreover, improvement is needed in MSC-EV targeting, which requires knowledge of disease mechanisms, forcing a better understanding before an EV treatment strategy can be developed. Additionally, the half-life and biodistribution of EVs in the body need further investigation and exploration through imaging techniques since these properties are affected by the different routes of administration. Furthermore, ncRNA cargo raises questions that remain unanswered, such as whether the simultaneous transfection of several miRNAs or cargos into MSC-EVs would be more effective or increase the side effects, or the potential effects of cell-free therapy depending on different pharmacological pre-treatments.

Last but not least, the effects of MSC-EVs, including exosomes, in RA, T1DM, and SLE remain unclear, although they have been demonstrated to possess similar biological effects as their parent MSCs. For this reason, despite their potential as in vivo transport carriers, before the application of MSC-EVs into clinical practice, further research with animal models and clinical assays needs to be addressed to test the safety and efficiency of this novel cell-free therapy for ADs.

## Figures and Tables

**Table 1:** Preclinical studies based on MSC-EV therapy in autoimmune diseases.

MSC Source	ncRNA Cargo	Disease Model	Admin. Way	Mechanism/Effect	Ref.
BM-MSCs	miR-150-5p	FLS and HUVEC in vitro cells; CIA mice model	IP	Modulates MMP14 and VEGF	[213]
BM-MSCs	miR-320a	In vitro and CIA mice model	IV	Regulates RA FLS activation by suppressing CXCL9 expression	[227]
BM-MSCs	miR-34a	RA FLS in vitro model and rat model	IV	Reduces inflammation via the cyclin I/ATM/ATR/p53 axis	[228]
BM-MSCs	miR-192-5p	CIA rat model	IA	Delays the inflammatory response	[218]
BM-MSCs	miR-124a	MH7A cell line	-	Inhibits proliferation and migration of FLS cell line and promotes apoptosis	[229]
BM-MSCs	lncRNA HAND2-AS1	Human synovial cell line MH7A	-	Impairs RA FLS activation through miR-143-3p/TNFAIP3/NF-κB pathway	[230]
BM-MSCs	circFBXW7	human synovial cell line and rat model	ID	Attenuates cell proliferation, migration and inflammation of FLS by targeting miR-216a-3p/HDAC4	[187]
Synovial-MSCs	Ad-circEDIL3	CIA mice model	IA	Downregulates the expression of VEGF induced by the IL-6/sIL-6R complex	[217]
BM-MSCs	miR-17, miR-23a and miR-125b	db/db diabetic mice	IV	Ameliorates peripheral neuropathy through TLR4/NF-κB signalling pathway	[231]
UC-MSCs	miR-126	STZ diabetic rats; HG-treated HRECs	IVT	Reduces retinal inflammation by downregulating the HMGB1 pathway	[219]
BM-MSCs	miR-21-5p	STZ diabetic rats and HG-treated CCSMCs	IV	Ameliorates erectile dysfunction through PDCD4 downregulation	[232]
AD-MSCs	miR-222	STZ diabetic rabbits	IV, SC and IO	Retina regeneration	[220]
AD-MSCs	miR-192	STZ diabetic rats	IVT	Relieves inflammatory response and angiogenesis ameliorating diabetic retinal damage through downregulation of ITGA1	[221]
BM-MSCs	miR-486-3p	HG-treated Muller cells	-	Inhibits oxidative stress, inflammation and apoptosis in diabetic retinopathy via TLR4/NF-κB axis repression	[233]
BM-MSCs	miR-125b	Kidney epithelial cells HG-treated	-	Induces autophagy and inhibition of apoptosis in diabetic nephropathy via downregulation of TRAF6	[234]
AD-MSCs	miR-125a	STZ diabetic rats and HG-treated rat glomerular mesangial cell (GMC)	IV	Protects against diabetic nephropathy by targeting Histone Deacetylase 1 and downregulating Endothelin-1	[235]
UC-MSCs	miR-21	Hypoxia on Beta cells (βTC-6)	-	Protects beta cells against apoptosis, alleviating ER stress and inhibiting p38 MAPK signalling	[236]
BM-MSCs	lncRNA SNHG7	HRMECs in vitro model of diabetic retinopathy	-	Suppresses endothelial-mesenchymal transition and tube formation trough miR-34a-5p-XBP1 axis	[181]
BM-MSCs	miR-146a	db/db diabetic mice	IV	Suppresses peripheral blood inflammatory monocytes and activation of endothelial cells via inhibiting Toll-like receptor (TLR)-4/NF-κB signalling pathway in peripheral neuropathy	[237]
UC-MSCs	miR-let-7b	STZ diabetic rats	Topical	Macrophage polarization and resolution of chronic inflammation for wound healing	[209]
Myeloid-derived MSCs	lncRNA H19	STZ diabetic mice	SI	Promotes wound healing in diabetic foot ulcers by upregulating PTEN via miR-152-3p	[208]
MSC	tsRNA-21109	THP-1 cells differentiated to macrophages	-	Alleviates SLE by inhibiting macrophage M1 polarization	[238]

AD-MSCs: adipose-derived mesenchymal stem cells; BM-MSCs: bone marrow-derived mesen-chymal stem cells; UC-MSCs: umbilical cord-derived mesenchymal stem cells; CCSMCs: corpus cavernosum smooth muscle cells; human retinal endothelial cells (HRECs); HRMECs: Human retinal microvascular endothelial cells; HG: high glucose; RA: rheumatoid arthritis; FLS: fibro-blast-like synoviocytes; STZ: streptozotocin-induced; CIA: Collagen-Induced Arthritis; IP: intra-peritoneal; IV: intravenous; IVT: intravitreal; ID: intradermal; IA: intraarticular; SC: subconjunc-tival; SI: skin injection; IO: intraocular.

**Table 2:** Advantages and limitations of MSC and MSC-EVs for therapy in autoimmune diseases.

	MSCs	MSC-EVs
Advantages	Repair and regeneration of injured cells and tissues (i.e., cartilage, bone, skin…)	Maintainance of MSC regenerative potential
	Immunoregulatory properties modulating B cells, T cells, NK cells, DCs, promoting macrophage polarization, etc…	Effectors of MSC immunoregulatory properties
	Low immunogenicity	Avoidance of tumorgeneity in transplanted chondrocytes
		Suppression of toxicity and immunogenicity in target organs/tissues (peripheral nerves, joints, eyes, skin…)
		Absence of genetic mutability
		Modification of EV surface for targeting specific organs/tissues (i.e., skin, eye…)
		Allowing specific cargo loading for enhancing the regenerative power in wound healing, degenerated nerve or cartilage
		Increase of immunomodulatory properties by pre-conditioning MSCs to enhance quantity of secreted EVs
		Longer circulating half-life and more biocompatible compared to liposomes and polymeric nanoparticles
		High stability and resistance to freeze–thaw cycles
		Quick and effective sterilization
		Ability to cross the blood–brain barrier and freely circulate through the microvasculature
Limitations	Poor cell survival	Absence of standarized methods for characterization
	Immune rejection	Lacking scalable production and purification
	High cost production	Requiring an improvement in the targeting strategies
	Perpetuation of MSCs in the body after disease	Needing for a better knowledge of half-life biodistribution, side effects and mechanisms of action
	Loss of stemness induced by time/aging	
	Undesired differentiation that can produce ossification, calcification and tumorigenesis	
	Inability to cross the blood-brain barrier and trapping in organs such as liver or lung	

DC: dendritic cell; EV: extracellular vesicle; MSC: mesenchymal stem cell; NK: natural killer.
